# Study protocol for comparing Screening, Brief Intervention, and Referral to Treatment (SBIRT) to referral as usual for depression in African American churches

**DOI:** 10.1186/s13063-021-05767-8

**Published:** 2022-01-31

**Authors:** Sidney H. Hankerson, Rachel Shelton, Myrna Weissman, Kenneth B. Wells, Jeanne Teresi, Janhavi Mallaiah, Amita Joshua, Olajide Williams

**Affiliations:** 1 Columbia University Irving Medical Center, 1051 Riverside Drive, New York, NY 10032 USA; 2 Columbia University, Mailman School of Public Health, 722 West 168th Street, Room 941, New York, NY 10032 USA; 3 University of California Los Angeles, 10920 Wilshire Blvd., Suite 300, Los Angeles, CA 90024 USA; 4 Hebrew Home at Riverdale, 5901 Palisade Avenue, Bronx, NY 10471 USA; 5 Columbia University Irving Medical Center, 710 West 168th Street, New York, NY 10032 USA

**Keywords:** African Americans, Depression, Health disparities, Patient Health Questionnaire-9, Screening, Brief Intervention, and Referral to Treatment, Community-based participatory research, Hybrid type 1 effectiveness-implementation design, Cluster randomized controlled trial

## Abstract

**Background:**

Depression is a leading cause of disability worldwide. African American adults, compared to White adults, are half as likely to be screened for depression in primary care settings. Disparities in depression screening contribute to poor clinical outcomes, as African Americans with depression are more disabled and sicker longer compared to Whites. African American churches are trusted settings that provide access to supports for depression. Indeed, in the first study of its kind, the investigators found that 20% of adults in African American churches screened positive for depression using the Patient Health Questionnaire-9 (PHQ-9). However, no subjects with a positive screen (PHQ-9 ≥ 10) accepted a treatment referral when offered by research personnel. Community Health Workers, who are trusted paraprofessionals from the target community, may bridge the gap between depression screening and treatment. The investigators have trained and certified 112 Community Health Workers from 45 African American churches in New York City to deliver an evidence-based intervention called Screening, Brief Intervention, and Referral to Treatment (SBIRT). Thus, the aim of the current study is to test the impact of Community Health Worker-delivered depression screening in Black churches on engagement with clinical services.

**Methods:**

Using a hybrid type 1 effectiveness-implementation design, we propose a 2-arm, mixed-methods cluster randomized controlled trial. Church study sites will be randomized to either SBIRT (intervention arm) or referral as usual (usual care arm). This trial will be conducted with 600 church members across 30 churches (300 intervention; 300 usual care). Our primary outcome is treatment engagement, defined as attending a depression-related clinical visit. Secondary outcomes will be changes in Mental Health-Related Quality of Life and depressive symptoms at 3 and 6 months post-screening. Lastly, we will conduct a concurrent, mixed-methods (qualitative-quantitative) process evaluation to assess contextual facilitators and barriers of screening and referral.

**Discussion:**

This is the first randomized trial of a church-placed, community health worker-delivered intervention for depression in African American populations. This study may provide a novel and effective approach to increasing depression identification and treatment linkage in economically disadvantaged populations with high depression rates.

**Trial registration:**

ClinicalTrials.govNCT04524767. Registered on 21 August 2020.

## Administrative information

Note: the numbers in curly brackets in this protocol refer to SPIRIT checklist item numbers. The order of the items has been modified to group similar items (see http://www.equator-network.org/reporting-guidelines/spirit-2013-statement-defining-standard-protocol-items-for-clinical-trials/).

**Table:** 

Title {1}	Study protocol for comparing Screening, Brief Intervention, and Referral to Treatment (SBIRT) to referral as usual for depression in African American churches
Trial registration {2a and 2b}.	NCT04524767; clinicaltrials.gov; August 21, 2020
Protocol version {3}	IRB-AAAT1474; July 27, 2020
Funding {4}	This study is funded by the National Institute of Mental Health (1R01MH121590-01A1)
Author details {5a}	Sidney H. Hankerson, MD, MBA (corresponding author) Columbia University Irving Medical Center 1051 Riverside Drive New York, NY 10032 Email: Sidney.Hankerson@nyspi.colubmia.edu Phone: 646-774-6429 Fax: 646-774-6439 Rachel C. Shelton, ScD, MPH Columbia University, Mailman School of Public Health 722 West 168th Street, Room 941 New York, NY USA 10032 Myrna Weissman, PhD Columbia University Irving Medical Center 1051 Riverside Drive New York, NY 10032 Kenneth B. Wells, MD, MPH University of California Los Angeles 10920 Wilshire Blvd., Suite 300 Los Angeles, CA 90024 Jeanne Teresi, PhD, EdD Hebrew Home at Riverdale 5901 Palisade Avenue Bronx, NY 10471 Janhavi Mallaiah, MBBS, MPH Columbia University Irving Medical Center 710 West 168^th^ Street New York, NY 10032 Amita Joshua, MPH Columbia University Irving Medical Center 1051 Riverside Drive New York, NY 10032 Olajide Williams, MD, MS Columbia University Irving Medical Center 710 West 168^th^ Street New York, NY 10032
Name and contact information for the trial sponsor {5b}	Denise M. Juliano-Bult, MSW National Institute of Mental Health Email: djuliano@mail.nih.gov Phone: 301-443-1638 Fax: 301-443-4045
Role of sponsor {5c}	The study sponsor did not have any role in study design; collection, management, analysis, and interpretation of data; writing of the report; and the decision to submit the report for publication; nor will they will have ultimate authority over any of these activities.

## Introduction

### Background and rationale {6a}

Although African American (AA) adults are more disabled by depression and have more severe symptoms, they are less likely to receive treatment for depression compared to whites [1, 2]. While reasons for these disparities are multifactorial [3], the major rate-limiting step to treatment engagement remains entrenched in factors governing access to care and care-seeking behavior. Significantly, a dearth of evidence-based interventions targeting treatment engagement for depression among African Americans exists. Using a Hybrid Type 1 Implementation-Effectiveness design, we propose to test a cluster randomized church-placed behavioral intervention, mediated by resident CHWs, to increase identification and treatment of depression among AAs. While our primary goal is to establish the effectiveness of the intervention, our hybrid design will help us better understand cultural and contextual factors influencing treatment engagement and sustainability.

#### Racial disparities in depression treatment

Even after adjusting for income, education, and insurance coverage, AAs have less access to depression treatment compared to Whites [4]. In fact, one study showed that compared with 54% of non-Hispanic Whites, only 40% of AAs with depression in the past 12 months received any type of treatment [5]. Major contributors toward this treatment gap include limited access and under-detection, which have been associated with clinical misdiagnosis from a provider [6], distrust of providers [7], and socio-cultural factors related to low care-seeking behavior [8].

#### The problem of care-seeking behavior for depression

In addition to more structural-level analyses, it is critical to address cultural and contextual factors influencing care-seeking behaviors for depression treatment [9]. Examples of these factors among low-income AAs include losing pay from work, stigma and shame, mistrust of healthcare providers, reliance on faith for emotional support, and self-management [4]. Indeed, it is important to understand the cultural and cognitive variability in the initial expression and conceptualization of distress and local perspectives on depression treatment [10], which may help tailor behavioral interventions designed to improve and *sustain* depression treatment among African Americans. Conversely, the failure to understand these nuances may propagate the current status quo in which *AAs with depression who do seek treatment are more likely to terminate from treatment prematurely or be hospitalized* [11].

#### The challenge of screening for depression among African Americans

Given the disabling and pervasive nature of depression, the 2016 U.S. Preventive Services Task Force (USPSTF) recommends screening for depression in adults 18 years and older [12]. However, a nationally representative study found that AAs are half as likely to be screened for depression in primary care settings compared to white adults [13]. Numerous barriers contribute to these disparities in depression screening including stigma [14–16], distrust of providers [7], lack of insurance [17], and financial constraints [18]. Lack of screening and detection contribute to poorer clinical outcomes including greater disability from depression and have a longer illness course compared to Whites [2].

#### The Black church and depression

Given their history of volunteerism, social support, and role as emotional sanctuaries, Black churches are ideally suited to increase the uptake of depression screening [19] and serve as referral centers. Churches are pillars of AA communities and provide access to groups that are already convening regularly. AAs have the highest rates of church attendance and self-rated religious importance among all racial/ ethnic groups in the USA. In urban AA communities, 65–80% attend church regularly and 55% are involved in church-related activities [20]. A church “Health Ministry” is a committee of church volunteers who champion health-related activities [21]. Health Ministries have been used to implement church-based screening for cancer [22–24], diabetes [25–27], obesity [28–30], and heart disease [31–33], among others. AA clergy are regarded as trusted “gatekeepers” for providing brief depression counseling and referrals to mental health specialists [34]. Indeed, 72% of AAs with a serious personal problem, including depression, seek help from clergy in Black churches [35]. However, much of the published research in Black churches has focused on medical diseases [21], with only sparse data available on depression [36], and treatment engagement for depression.

### Objectives {7}

The overarching aim of the current study is to expand the scope of these CHWs to include depression screening, brief intervention, and referral. Using a Hybrid Type 1 Effectiveness-Implementation design [37], we propose a 2-arm, mixed-methods cluster randomized controlled trial within 30 Black churches our CHWs currently attend. Guided by the Consolidated Framework for Implementation Research (CFIR), we will assess key implementation variables related to depression screening uptake. We will also assess patient-level barriers and facilitators of help-seeking behaviors for depression. Our specific aims are as follows:

#### Aim 1: To compare the effect of SBIRT (intervention arm) to referral as usual (RAU) (usual care arm) on treatment engagement

We will randomize 15 churches to each study arm. Adults with a positive depression screen (*n* = 600) will receive either SBIRT (Screening + Brief Intervention + Referral to Treatment) or RAU (list of treatment sites + depression education pamphlets). We hypothesize that SBIRT will lead to increased treatment engagement (primary outcome) compared to RAU at 6 months post-screening.

#### Aim 2: To assess changes in mental health outcomes at 3 and 6 months post-screening

We hypothesize that adults in the SBIRT arm will have better Mental Health-Related Quality of Life and fewer depressive symptoms (secondary outcomes) compared to those in the RAU arm.

#### Aim 3: To identify contextual factors that act as facilitators or barriers of depression screening and referral

Guided by the Consolidated Framework for Implementation Research (CFIR) [19], we will conduct a mixed-methods process evaluation with key stakeholders (clergy, CHWs, and congregants with positive PHQ-9 screen) to understand multilevel influences on depression screening and referral.

### Trial design {8}

**Fig. 1:**
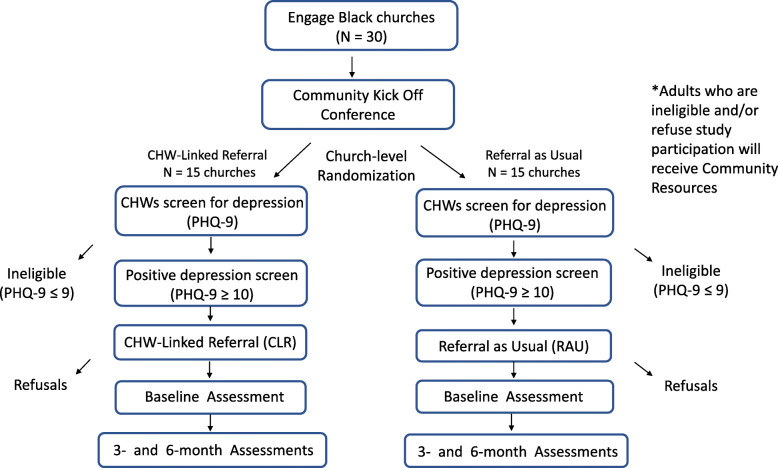
Randomization of churches

Using a Hybrid Type 1 Effectiveness-Implementation design [37], we will conduct a two-arm, mixed-methods cluster randomized controlled trial within 30 Black churches our CHWs currently attend. Churches will be randomized in a 1:1 ratio to either SBIRT or RAU (Fig. 1). This is a superiority study design that takes a dual focus in assessing clinical effectiveness and implementation. The effects of the intervention (SBIRT) will be tested on relevant outcomes while observing and gathering information on implementation. The primary outcome is treatment engagement for depression (clinical encounters). Secondary outcomes include the QIDS-SR, the PROMIS Depression scale, and the SF-12 measure of quality of life (QoL). Guided by the Consolidated Framework for Implementation Research (CFIR), we will assess key implementation variables related to depression screening uptake. We will also assess patient-level barriers and facilitators of help-seeking behaviors for depression. Data collection will be prospective. We will randomize 15 churches to the intervention arm (SBIRT) and 15 churches to the control arm (referral as usual).

## Methods

### Study setting {9}

This study will be conducted in 30 Black churches in New York City [38]. Churches with a mean congregation size of at least 500 members where CHWs currently attend will be recruited. We have obtained letters of support from these 30 churches plus additional church networks to provide a recruitment buffer pool.

### Eligibility criteria {10}

#### Eligibility for general depression screening

Inclusion criteria are as follows: (1) adults 18 years and older and (2) English speaking. There are no exclusion criteria for general screening.

#### Eligibility for participation in RCT among subjects who screen positive

Inclusion criteria are as follows: (1) adults 18 years and older; (2) English speaking; (3) PHQ-9 ≥ 10. Exclusion criteria are reporting active suicidality, or verbally endorsing homicidal ideation or psychotic symptoms, and those actively receiving formal treatment for depression or mental health illness (e.g., with medications and/or psychotherapy).

### Informed consent {26a}

The Project Coordinator(s) (PC) will focus on consenting eligible adults who screen positive for the randomized controlled trial. When the prospective participant has completed the PHQ-9, the Community Health Worker and PC will briefly calculate their total PHQ-9 score and assess for suicidality. Individuals who score 10 on the PHQ-9 will be contacted within 14 days to schedule a baseline assessment and verify study eligibility. A telephone interview assessment will then be scheduled for those who agree. At the start of this interview, the interviewer will obtain informed consent from the patient. The consent will include permission to contact the participant’s treatment facility where they seek depression treatment, obtain selected data from the patients’ other health care providers, and share information with our CUIMC collaborators. All participants will be informed that they can refuse to answer questions, stop the assessment at any time, or withdraw from the study if they so wish, without in any way affecting their eligibility or receipt of usual health or social services.

### Additional consent provisions for collection and use of participant data and biological specimens {26b}

There are no additional consent provisions. We will not be collecting biological specimens.

## Interventions

### Explanation for the choice of comparators {6b}

We will compare the effectiveness of CHW-delivered Screening, Brief Intervention, and Referral to Treatment (SBIRT, *n* = 15 churches) to referral as usual (RAU; *n* = 15 churches) on treatment engagement (primary outcome). SBIRT is an evidence-based approach designed to provide screening, brief intervention, and referral to more intensive treatment for people at risk of developing mental disorders, including depression [39, 40]. Distributing depression educational materials and contact information for treatment providers is the most common form of referral. This practice will represent “usual care” for our study.

### Intervention description {11a}

#### The study intervention: Screening, Brief Intervention, and Referral to Treatment (SBIRT)

SBIRT is composed of three core components: screening with a validated instrument, brief intervention, referral to treatment [39].

The Patient Health Questionnaire-9 (PHQ-9) is the most widely utilized depression screening instrument in primary care settings. The PHQ-9 is a brief, valid, and reliable measure that tabulates frequency of DSM-IV depressive symptoms in the prior 2 weeks [41–43].

It has been shown to be valid and reliable among AAs and other racially diverse clinical and population samples [44–46].

Motivational interviewing (MI) is the brief intervention most commonly used in SBIRT. MI is an empirically tested, person-centered, behavior change intervention designed to guide, elicit, and strengthen motivation for change [47]. MI is an effective preparatory intervention that decreases ambivalence about therapy and increases motivation for more intensive treatment [48, 49].

The final component of SBIRT involves actual referrals to treatment. This begins with determination of the individual’s health insurance status. Persons without insurance will be enrolled with the assistance of CHWs (who are certified New York State Insurance Navigators) into New York State health plans (insurance exchange or Medicaid). Based on our preliminary studies, we expect 12 to 15% of subjects to be uninsured. Individuals ineligible for health insurance will be referred to our network of free clinics providing mental health services. Uninsured individuals will also be referred to the NYC Dept. of Health Action Centers, to insurance information, and other social services. Referral involves the CHW calling (using study-issued prepaid calling cards) or offering to call the provider’s office on behalf of the subject to make an appointment, plus a follow-up appointment call reminder the day before the appointment.

CHWs will provide two MI sessions in each of the first 3 months, for a maximum of five MI sessions over 3 months (Table 1). The initial two sessions will be conducted in person at the church and follow-up sessions will occur either in church or over the phone. Data will be collected on method of delivery. MI sessions will create a nonjudgmental and supportive environment for eligible participants to move through the various stages of change associated with depression help-seeking. In the initial session, CHWs will focus on establishing rapport using open-ended questions, affirmations, reflections, and summary statements (OARS). They will then review depression symptoms based on the participants PHQ-9 score. The second session will focus on assessing motivation and confidence in seeking treatment and elicit barriers and facilitators for depression treatment. Follow-up sessions will involve summarizing the “pros” and “cons” of depression treatment; providing options for the participant based on the nature of barriers elicited from them; assessing participant’s values and goals, to help them link their current mental health pattern to their goals; and summarizing what was discussed to clarify an action plan.

**Table 1:** CHW Depression Booster Sessions

Module	Topics covered
Screening for Depression	Scoring the PHQ-9, impact of depression on physical health, study procedures
Safety Assessment and Planning	Scoring the C-SSRS, Stanley-Brown Safety Plan documentation
Mental Health First Aid	Mood, anxiety, psychotic, and substance use disorders, 5-step action plan to assist in crisis
Trauma	Overview of Adverse Childhood Experiences (ACEs), PTSD, talking with traumatized adults
Treatment Referral Sites	Mental Health Community Resource Directory, navigating access barriers
Human Subjects	Collaborative Institutional Training Initiative (CITI) and HIPAA

#### The control arm: referral as usual

We will utilize depression educational brochures describing the nine hallmark symptoms of depression symptoms and the importance of seeking treatment: one from the National Institute of Mental Health (NIMH), one from the American Psychiatric Association (APA), and one from National Alliance of Mental Illness (NAMI). CHWs assigned to usual care churches will distribute the list of referral sites and pamphlets to study participants at designated screening events. No specific referrals will be made in this arm.

#### Virtual study procedures

Considering COVID-19 and the New York State on Pause law, we will also conduct study procedures virtually. Study Project Coordinators will facilitate Columbia-HIPPA compliant Zoom screening events with church members from approved church study sites. The same study procedures outlined above will be followed; however, the interactions will be conducted via Zoom or telephone.

### Criteria for discontinuing or modifying allocated interventions {11b}

Educational pamphlets will be reviewed by the Community Coalition Advisory Board and modified as needed for cultural relevance to target population.

### Strategies to improve adherence to interventions {11c}

Our strategies to improve adherence are innovating in several ways:

1. *Strategy of the intervention.* We will use an evidence-based approach called Screening, Brief Intervention, and Referral to Treatment (SBIRT), which is composed of three core components: screening with a validated instrument, brief intervention, and referral to treatment. To our knowledge, ours would be the first study to test the effectiveness of SBIRT for depression in Black churches.
2. *Church-placed setting.* Although most depression screening studies have been conducted in primary care settings, AAs are half as likely to be screened for depression in primary care settings compared to white adults. We have therefore adopted a “meet people where they are” approach by integrating our intervention into the church setting where its implementation will occur.
3. *Use of Community Health Workers.* The influence of mistrust in care-seeking behavior for depression cannot be overstated in AA communities. To overcome this barrier, CHWs—trusted members of the local community—recruited and trained from churches where our intervention will take place, will be responsible for delivering the SBIRT. CHWs will conduct one-on-one sessions with participants to deliver the intervention or control arms, and thus will be able to monitor adherence. In addition, each CHW will collaborate with church leaders to distribute a virtual recruitment video to generate interest within the church membership.

### Relevant concomitant care permitted or prohibited during the trial {11d}

Exclusion for participation in RCT among subjects who screen positive include reporting active suicidality, or verbally endorsing homicidal ideation or psychotic symptoms, and those actively receiving formal treatment for depression or mental health illness (e.g., with medications and/or psychotherapy).

### Provisions for post-trial care {30}

Due to the classification of this study as minimal risk, we do not anticipate participants will suffer from harm due to trial participation. There is no compensation to those who suffer harm from trial participation.

### Outcomes {12}

The primary outcome is treatment engagement for depression (clinical encounters), defined as attending a depression-related clinical visit for which the subject reported receiving information, referral, counseling, or medication for depression. Treatment engagement will be assessed by the Community Partners in Care (CPIC) Health Services Use Survey (NIMH R01 MH078853 – Wells PI) at 3 and 6 months post-screening. Secondary outcomes include mental health-related quality of life and depressive symptoms at 3 and 6 months post-screening. We will also identify contextual factors that act as facilitators or barriers of depression screening.

### Participant timeline {13}

### Sample size {14}

The design is a cluster randomized trial with randomization at the level of churches. There will be 30 churches, 15 randomized to the intervention (SBIRT) and 15 to the usual care arm (referral as usual). We will screen an average of 100 adults per church, yielding a pool of 3000 respondents. Based on our pilot study, we posit that 20% of church attendees will screen positive for depression, defined as PHQ-9 ≥ 10. The primary outcome is treatment engagement for depression (clinical encounters). Secondary outcomes include the QIDS-SR (mean = 16.3, SD = 4.0 estimated from a previous study in an ethnically diverse sample) [50], the PROMIS Depression scale (mean = 50, SD = 10), PROMIS, and the SF-12 measure of quality of life (QoL) (mean = 50 and SD = 10). Clinically meaningful change on the QIDS-SR has been estimated as about 5 points. For the PROMIS short-form measure, a minimally important difference has been reported as an effect size of 0.3 to 0.5 (about one third to one half standard deviation units). There will be three waves of data: baseline, 3 months, and 6 months. Power calculations are presented for the primary outcome, and three outcome variables for which there are large amounts of data from previous studies of racially and ethnically diverse groups. 
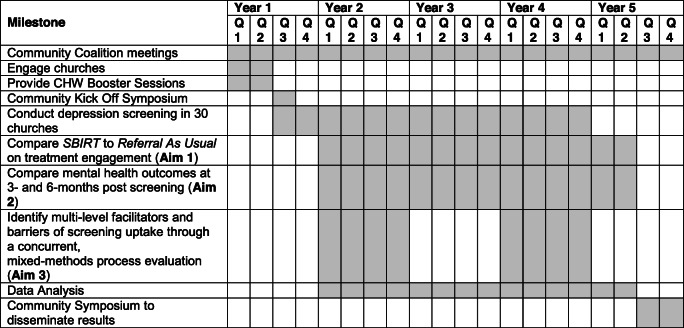


### Recruitment {15}

#### Recruitment of participants

This study involves three distinct study populations including CHWs (*n* = 60), adults screened with the PHQ-9 (*n* = 3000), subjects randomized to intervention or control conditions (*n* = 600), and clergy (*n* = 30).

#### Recruitment of community health workers

A CHWs from Columbia's InTOuCH Training Program will serve as interventionists in this study. A total of 60 certified CHWs (two per church) will be identified from the existing cohort of 112 members. note that our center trains 45 CHWs per year on an ongoing basis, which will further expand our recruitment pool of CHWs within each church at the time of project start date, if this proposal is successful. CHWs who express interest in the study will be given a formal interview that includes standardized questions covering the following topics: (1) clinical scenarios with depression; (2) experience and comfort working with people with depression/suicidality; (3) role clarity and expectations; (4) reasons for participation. Any CHW who was hospitalized for a medical or psychiatric condition in the preceding 6 months will be excluded.

#### Recruitment of community members

In total, 100 adults per church will be screened with the PHQ-9 yielding a pool of 3000 respondents. Church members who screen positive for depression with the PHQ-9 will be eligible to participate in the RCT (*n* = 600). CHWs will collaborate with church leaders to organize depression screening events at church health-focused programs. Additionally, funds will be provided for one screening event to take place as part of a Church Mental Health Forum. Two CHWs and at least one Project Coordinator (PC) will be present at the church for each screening event to assist with scoring the PHQ-9, interpreting the results, and study enrollment. We have successfully used mental health forums and health fairs to recruit participants in our church-based depression screening study and other church-based trials. Church members will also be recruited virtually via Zoom as described in the study procedures.

#### Recruitment of clergy

The lead pastor of each church study site will be recruited for participation in a semi-structured interview. The purpose of the interview is to explore clergy’s perspectives of church-based depression screening and awareness of CHW activities in their church. Clergy will receive a $100 gift card as compensation in the interview.

## Assignment of interventions: allocation

### Sequence generation {16a}

Randomization will occur at the church level to prevent contamination and other threats to internal validity through interactions between members of the same church. The study statistician will develop algorithms for conducting randomization that take into account clustering of churches matched on number of members and rolling enrollment. This randomization procedure will be carried out using SAS macro *after church members complete the baseline interview.* Churches will be grouped to K categorical groups according to church size, for example, small, medium, large. A random number from 0 to 1 will be used to determine the assignment group. The standard cut score will be set at 0.5 for the first *n* churches within each categorical church size. Churches that receive a random number between 0 and 0.5 will be assigned to the RAU group and those with a random number greater than 0.5 will be assigned to the intervention group. The balance between the groups within each categorical church size will be carefully weighted after the total number of churches from a group reaches a number greater than *n*. Before the randomization procedure, the number of churches randomized to each arm will be estimated using SAS macro programs. If more than the *n* churches are randomized initially, the cut score for the next church is equal to the ratio of the intervention group (*n*1) to the churches already randomized (*m*) for that group (*n*1/*m*). For example, if there will be 30 churches for randomization and the *n* is set to 5, the first five facilities (*n*) will be randomized to the standard cut score 0.5 (about half will go to the intervention and half to the RAU group). The sixth church’s randomization cut score is equal to the number of churches in the intervention group (*n*1) divided by the total number of churches randomized within that group (in this case, the denominator *m* is 5.) The seventh church’s cut score will be adjusted according to the previous six churches, and so forth.

### Concealment mechanism {16b}

The biostatistician who runs the randomization procedure passes the information directly to the Project Director who alerts the churches of their assignment. The principal investigator who oversees the data coordinating center (DCC) will be the only other person made aware of the assignment.

### Implementation {16c}

Two CHWs and at least one Project Coordinator (PC) will be present at the church for each screening event to assist with scoring the PHQ-9, interpreting the results, and study enrolment.

Within 2 weeks of completing the PHQ-9 and study enrollment, Project Coordinators will conduct telephone baseline assessments with participants who sign informed consent.

Following baseline data collection, churches will be randomized to either the intervention (SBIRT) or usual care arm (referral as usual) arm. The randomization sequence will be generated by the study statistician at a site away from the churches, in accordance with the CONSORT guidelines.

## Assignment of interventions: blinding

### Procedure for blinding {17a}

The research personnel who will be collecting data at baseline, 3 months, and 6 months will be blinded.

### Procedure for unblinding if needed {17b}

There are no circumstances where unblinding would be needed.

## Data collection and management

### Plans for assessment and collection of outcomes {18a}

We will be utilizing the Patient Health Questionnaire-9 (PHQ-9) to screen participants for depression. Depressive scores between 0 and 9 indicate “no depression” or “mild” symptoms, those between 10 and 19 are rated as “moderate” and “moderately severe,” and 20 or greater as “severe.” A score of PHQ-9 ≥ 10 is recommended as the single cutoff point for depression, with a sensitivity and specificity of 88% [51].

A two-page screening survey will include the PHQ-9, demographic information, literacy level assessment, and depression treatment history / preferences [52]. Participants will submit their completed survey to the CHW in a private church space, who will quickly assess for suicidality by examining responses to Question #9 of the PHQ-9. Eligibility for the RCT depends on the participants’ total PHQ-9 score:

- Participants whose PHQ-9 ≤ 9 (negative depression screen), are ineligible for the RCT and will receive a copy of the CHW Community Resources guide.
- Participants whose PHQ-9 ≥ 10 (positive depression screen) will be eligible for the RCT and invited to sign informed consent to participate in the study.

Within 2 weeks of completing the PHQ-9 and study enrollment, Project Coordinators will conduct telephone baseline assessments with participants who signed informed consent. The Mini-International Neuropsychiatric Interview (MINI) [53] will be used to assess criteria for DSM-5 psychiatric disorders. Table 2 shows the name and construct for each clinical measure collected at baseline, 3 months, and 6 months post-screening.

**Table 2:** Clinical measures

	Baseline	Months	
		3	6
Demographic characteristics (e.g., Race, Income, Education)	x		
Mini-International Neuropsychiatric Interview (MINI)	x		
PhenX Social Determinants of Health (e.g., Food Insecurity)	x		
CPIC Health Services Use Survey (1° outcomes)		x	x
Mental Health Provider Appointment Attendance Verification Form		x	x
12-Item Short-Form (Mental Health-Related QoL – 2° outcome)		x	x
16-Item Quick Inventory of Depressive Symptoms (2° outcome)	x	x	x
NIH-PROMIS Depression Scale (2° outcome)	x	x	x
Patient Health Questionnaire-9 (Depressive severity)	x	x	x
Charlson Comorbidity Index (Medical Comorbidity)	x	x	x
Depression Stigma Scale (Mental Health Literacy / Stigma)	x	x	x
Multidimensional Measure of Religiosity (Religious Involvement)	x	x	x
Medical Outcomes Social Support Scale (Social Support)	x	x	x
Everyday Discrimination Scale	x	x	x

#### Confirmation of treatment engagement (primary outcome)

Treatment engagement will be assessed by the Community Partners in Care (CPIC) Health Services Use Survey (NIMH R01 MH078853 – Wells PI), which has been validated in multiple studies with AAs [54–59]. We will also attempt to directly confirm treatment engagement from the mental health provider’s clinic. The latter will be conducted by phone during a conversation between RAs and mental health clinic staff at 3 and 6 months. Participating subjects will be given a personal identification number that will be entered into a HIPAA compliant form (Mental Health Provider Appointment Attendance Verification Form) by Project Coordinators during the clinic confirmation phone call. We have obtained letters of support from local provider networks from whom we will confirm whether the client did or did not attend the mental health visit, although we will not be collecting PHI on the clinical details of the visit. CHWs in both the Intervention and Usual Care arms will maintain a log of study subject information using HIPAA compliant procedures. These logs will contain the subjects’ name, date of birth, date and results of PHQ-9 screening, and the name, address, and phone number of the provider to whom the subject was referred. At the subject level, we will also include treatment engagement assessments in follow-up surveys at 3 and 6 months. These subject-level surveys will include transtheoretical model questions probing the stages of change with regard to depression treatment-seeking behaviors.

#### Assess change in mental health outcomes at baseline, 3 months, and 6 months post-screening (secondary outcome)

At baseline, RAs will administer the Mini-International Neuropsychiatric Interview (MINI), a brief structured interview used to diagnose major psychiatric disorders in DSM-5 and ICD-10. Additionally, the social determinants of health (SDOH) will be assessed using the NIMHD PhenX SDOH toolkit at baseline. Participants will be contacted by RAs at 3 and 6 months for a follow-up telephone assessment. Mental Health-Related Quality of Life (QoL) will be assessed by the 12-Item Medical Outcomes Study Short-Form (SF-12) [60]. Depressive symptoms will be assessed by the 16-Item Quick Inventory of Depressive Symptomatology (QIDS-SR) [61] and the NIH-PROMIS-Depression Scale [62].

#### Identify contextual factors that act as facilitators or barriers of depression screening uptake (secondary outcome)

We will employ mixed-methods research methodology to gather multilevel factors that influence screening uptake and treatment engagement. The Consolidated Framework for Implementation Research (CFIR) [40] from the field of implementation science will guide our exploration. Data collection and analysis will proceed concurrently (QUAN+QUAL)^87^ to understand effectiveness and implementation outcomes in a more nuanced fashion. First, we will use the CFIR as a guide to conduct semi-structured interviews across each level of the Socio-Ecological Model: individual level [randomly selected depression positive subjects (*n* = 60)], interpersonal level [CHWs (*n* = 60)], institutional level [clergy from each site (*n* = 30)]. Each pastor will also complete the Faith-Based Organization Capacity Inventory (FBO-CI) [63] interview to assess their church’s health promotion experience and research capacity. Findings from these interviews targeting multilevel barriers and facilitators across key stakeholders will be used to inform a future Hybrid Type 3 Implementation-Effectiveness trial. We will also collect implementation data on adoption—The percentage of churches that employ three Depression Screening programs in a year; and maintenance—The extent to which Depression Screening continues to be delivered 1 year following the end of the data collection period by CHWs in the absence of direct research support. Finally, to gain a deeper understanding into barriers and facilitators governing depression help-seeking behaviors, we will conduct focus groups and semi-structured interviews with subjects who *did and did not* seek treatment for depression from both the usual care *and* intervention groups.

Semi-structured interviews will be conducted either in person or over the telephone. Focus groups will occur at Columbia University’s Wellness Center. All interviews will be audiotaped, professionally transcribed and uploaded into an NVivo data file for qualitative analysis. Quantitative measures will be administered at church sites in person and over the telephone.

Semi-structured interviews and focus groups will be audiotaped with digital recorders and professionally transcribed. Clean transcripts will be uploaded into NVivo, a qualitative analysis software program that aids in the storage, organization, and retrieval of qualitative data. Thematic analysis will be used to analyze qualitative data [64]. The Co-Investigator of this study (Co-I), who is an expert in qualitative data collection and analysis, will train Masters-level Project Coordinators (PCs) in qualitative methods and oversee qualitative analysis. RAs will independently read all transcripts and develop an open coding schema based on a priori and emergent themes. They will present their coding schemes, discuss emergent themes, refine codes, and develop a final codebook that consists of a list of categories and topics. We will then use NVivo to code all data consistent with the codebook. To establish coding reliability, both the Co-PI and the Senior Program Manager will independently code up to 10% of the transcripts and identify kappa’s. Research staff will meet weekly during this process to review definitions and assignment of codes and resolve differences through consensus by checking the segment of transcript in question. Once all data have been coded based on the initial codebook, codes most relevant to the research questions will be subject to further analysis, with the goal of identifying overarching themes.

### Plans to promote participant retention and complete follow-up {18b}

#### Plan for church retention

Each church will receive a $1000 donation in appreciation for their time and space. Pastors will sign a *Memorandum of Understanding* that commits their church to (1) identify a church champion to assist CHWs implement screening days and (2) host at least 3 depression screenings per year.

#### Plan for CHW retention

We have developed and tested a retention program for CHWs—the Columbia University CHW Alumni program. This has led to an 85% retention of CHWs and is built around three pillars: (1) social engagement and relationship building; (2) continuous public health education; and (3) mindfulness training. These activities support CHWs in their role as community health paraprofessionals and builds self-efficacy.

#### Plan for participant retention

We will also implement additional retention strategies that have led to successful recruitment and retention of ethnic minorities in clinical trials such as regular telephone contact to remind participants of upcoming study appointments; toll-free study telephone number to report change of address and contact study staff; ethnic diversity of staff; and assistance with transportation to study visits via subway and bus passes [62–70].

We will attempt to collect all outcome measures from participants who discontinue. We will make phone calls to participants who drop out/withdraw.

### Data management {19}

This study has partnered with data coordinating center (DCC), an organization that is experienced in coordinating clinical trials and will be responsible for the following activities: (1) development of the computer-assisted data collection system, (2) staff training and certification in data collection, (3) randomization procedures, (4) data monitoring and quality control, (5) data processing, and (6) data analysis. The DCC will prepare regular reports for internal and external monitoring of progress toward study milestones and provide blinded and unblinded data requests.

All screening data will be collected using a form developed in REDCAP; evaluation data will be collected using a computer-assisted personal interview (CAPI) system. These methods provide accuracy in data collection, because the systems do not accept out-of-range values, and do not allow for deviation from prescribed skip patterns. The DCC will create scoring and cleaning programs for scales within instruments. Although the REDCAP and CAPI data entry systems should not allow these types of errors, the cleaning programs serve to double check the accuracy of the data. Periodically, the data manager will review all data for duplicate records, illogical collection dates or times of interview, outlier and out of range values, and illogical contingencies using program syntax created for each data file. After any corrections are made, items distributions will be reviewed to make sure no anomalies remain. In addition, the project coordinator periodically reviews entire files as a quality assurance measure.

Data storage, data safety, and security: Secure laptops and office desktop computers that are password protected and encryption enabled will be used for data collection. Laptops with encrypted data are and log sheets will also be kept in a locked onsite storage area.

At the DCC, electronic data are backed up daily or weekly to a backup server depending upon the receipt of data. Additional backup external hard drives are stored in a fireproof safe. Protected Health Information (PHI) is confined to a secure device that is not connected to the internet. All computers are password protected and the whole drive is encrypted with Bitlocker encryption. They are on a non-routable LAN network. No file and database servers are accessible to the public through the Internet. A hardware-based firewall device protects the network system against hackers and any unauthorized internet access. Spam and email filtering is built-in within the firewall device. The anti-virus software (McAffee Anti-Virus) protects the network from threats of viruses, worms, and Trojan horses and other malwares contained in email attachments and also from files downloaded through the internet. Through “push-technology,” this anti-virus software is automatically updated for all virus definitions and other updates.

### Confidentiality {27}

#### Patient information

Confidentiality of patient information will be safeguarded in several ways. All research staff will be thoroughly trained in the need to maintain strict confidentiality. Data will be reported in aggregate form only. All participant-identifying information on paper will be kept in locked files accessible only to study staff. All electronic information will be password protected. No information obtained during the study will be used for any purpose other than the purpose for which the person has consented. The physical risks of the study procedures are minimal and the potential risks have been outlined above. The clinical care of any given participant will be handled entirely by the participant’s primary care provider, unless emergency care is needed due to suicidality as outlined above, and study participants will be made aware of this at the baseline assessment. Similarly, any medical problem that arises during study visits will be referred to the participant’s primary care provider. For those who do not have any provider, the PC will provide the participant with information to local health center. Data will be collected in two ways: (1) paper assessments collected in person; (2) electronic assessments collected via Zoom will be collected via a computer-assisted personal interview (CAPI) program called The Survey System.

#### Paper assessments

To address confidentiality of paper assessments, hard copies of the completed assessments will be stored in a locked file at Data Coordinating Center for 10 years after study completion.

#### Electronic assessments

To address confidentiality of electronic assessments, data are stored directly on the computer hard drive and are not transmitted via the internet. In addition, the internet connectivity is disabled from study computers.

#### Secure data transfer procedures

As has been done with several other NIH projects, the statisticians at the Data Coordinating Center will download assessment data from the Columbia secure server through secure VPN connections.

#### Data access

There is a double layer of protection in order to access the data. First, they must login to the University VPN, then login again to the Columbia file server. Passwords for login are updated every 6 months. In addition, research personnel will be required to use password protected laptops/computers. Data will be stored on HIPAA compliant, encrypted servers that are only accessible to IRB-approved personnel.

Additional efforts to protect participant confidentiality to the extent permitted by law will be ensured by the following:

- Each study participant will receive a code number through which all study data will be linked. The code will only be known by research personnel.
- Participant names, code numbers, and study data will be kept in a single locked file, accessible only to key study personnel working on the study.
- Information stored on the computer will be coded numerically.
- All study data will be reported in tabular/group format while no individual data will be reported.
- Records will only be available to research staff, and the Federal, State, and Institutional regulatory personnel, who may review records as part of routine audits.
- Legal advocacy organizations that have the authority under state law can access confidential subject records, but cannot re-disclose this information without participant consent.

### Plans for collection, laboratory evaluation, and storage of biological specimens for genetic or molecular analysis in this trial/future use {33}

This study protocol does not include the collection of biological specimens.

## Statistical methods

### Statistical methods for primary and secondary outcomes {20a}

The design is a cluster randomized trial with randomization at the level of churches. There will be 30 churches, 15 randomized to the intervention (SBIRT), and 15 to the usual care arm (referral as usual). We will screen an average of 100 adults per church, yielding a pool of 3000 respondents. Based on our pilot study, we posit that 20% of church attendees will screen positive for depression, defined as PHQ-9 ≥ 10. The primary outcome is treatment engagement for depression (clinical encounters). Secondary outcomes include the QIDS-SR (mean = 16.3, SD = 4.0 estimated from a previous study in an ethnically diverse sample) [50], the PROMIS Depression scale (mean = 50, SD = 10), PROMIS, and the SF-12 measure of quality of life (QoL) (mean = 50 and SD = 10). Clinically meaningful change on the QIDS-SR has been estimated as about 5 points. For the PROMIS short-form measure, a minimally important difference has been reported as an effect size of 0.3 to 0.5 (about one third to one half standard deviation units). There will be three waves of data: baseline, 3 months, and 6 months. Power calculations are presented for the primary outcome, and three outcome variables for which there are large amounts of data from previous studies of racially and ethnically diverse groups.

#### Primary outcome: statistical approach and power for examination of endpoint differences, treating treatment engagement (clinical encounters) as binary

The most conservative model examines study endpoint differences using the methods of Fleiss [65]. A clinically important effect size of the treatment engagement (clinical encounters) rate is a difference of 5 to 20% between the usual care and intervention groups. Based on our prior studies the intra-cluster correlation coefficient (ICC) for church was 0.03. We assume that treatment engagement (clinical encounters) is measured with error (reliability = 0.95). With cluster randomization, the variance inflation factor (*V*_ifc_) can be calculated as:*V*_*ifc*_ = 1 + (*n*_*c*_ − 1)*p*_*c*_), where *n*_c_ is average number of subjects within church, *ρ*_c_ is the ICC within church. The sample size per group is: $m\ast ={\left({z}_{\alpha}\sqrt{2\overline{P}\overline{Q}}+{z}_{\beta}\sqrt{p_0{q}_0+{p}_1{q}_1}\right)}^2/{\left({p}_1-{p}_0\right)}^2$ [1], and adjusting by the continuity correction, reliability and cluster size, the sample size per group is: m = (m^∗^ + 2/(P_1_ − P_0_))V_ifc_/R where *V*_ifc_ is the variance inflation factor (VIF) and *R* is the reliability estimate.

Setting *α* = 0.05 for a two-tailed test, and adjusting for unreliability (*R* = 0.95) and clustering (*V*_ifc_ = 1.57 with *n*_c_ = 20, ICC_church_ = 0.03), the end point study difference detectable for power of 0.80 is 10.9% (assuming 9.1% in the usual care group, 20% in the intervention group). With an anticipated intent-to-treat (ITT) sample size of *m* = 300 subjects per group (assuming about 20 respondents per church and 15 churches per group), power will be adequate to detect clinically meaningful group differences in proportions. Even without ITT, assuming a worst-case scenario of 20% overall attrition, the resulting sample sizes of 240 per group will still permit detection of relatively small effect sizes of about 12.1% group differences in treatment engagement*.*

Examining all waves of data, and potential covariates due to imbalance, a logistic regression approach for binary outcome variables can be used. The model is: log(p/(1 − *p*)) = *β*_0_ + *β*_1_*x*, where *p*_0_ = Prob(*Y* = 1│|*x* = 0), *P*_1_ = Prob(*Y* = 1│*x* = 1), where *Y* is the treatment engagement outcome and *x* is a dummy variable for treatment group. SAS Proc GLIMMIX will be used to adjust for the design feature of clustering within churches. The total sample size for the logistic regression model is: ${N}^{\ast }=\frac{{\left(V{(0)}^{1/2}{Z}_{1-\alpha /2}+V{\left({\beta}^{\ast}\right)}^{1/2}{Z}_{1-\beta}\right)}^2}{P_1{\beta}^{\ast 2}}\left(1+2{P}_1\delta \right)$ [66, 67] with ${\beta}^{\ast }=\log \frac{P_2\left(1-{P}_1\right)}{P_1\left(1-{P}_2\right)}$, $V(0)\frac{1}{1-B}+\frac{1}{B}$, $V\left({\beta}^{\ast}\right)=\frac{1}{1-B}+\frac{1}{B\kern0.5em \exp \left({\beta}^{\ast}\right)}$, *δ* = (*V*(0)^1/2^ + *V*(*β*^∗^)^1/2^*R*)/(*V*(0)^1/2^ + *V*(*β*^∗^)^1/2^), *R* = *V*(*β*^∗^)*B*(1 − *B*)  exp(2*β*^∗^)/(*B*  exp(*β*^∗^) + (1 − *B*))^2^ where *P*_1_, *P*_2_ are the event rate at *x* = 0 (RAU group), and *x* = 1 (intervention group), *B* is the proportion of the sample with *x* = 1, adjusting for unreliability, multiple covariates (VIF, where *ρ*_x_ is max correlation between *x*, the group variable and other covariates), clustering (*V*_ifc_ = 1.57 (with *n*_c_ = 20, ICC_church_ = 0.03)) and longitudinal repeated measurement (variance inflation factor, *V*_*ifR*_ = 1 + (*T*_*n*_ − 1)*p*_*y*_)/*T*_*n*_ with *T*_*n*_ = 3): $N=\frac{N^{\ast }{V}_{ifc}{V}_{ifR}}{R_{el}\left(1-{\rho}_x\right)}$. The following table (Table 3) is for *α* = 0.05, 1 − *β* = 0.80 (power), *ρ*_x_ = 0.4 (adjusted for multiple covariates), *R*_el_ = 0.95(adjusted for reliability), *V*_ifc_ = 1.57 (cluster adjustment), *P*_1_(*X* = 0,control group), *P*_2_(*X* = 1,intervention group), *B* = 0.5, *ρ*_y_ = 0.5 and total sample size *N*:

**Table 3:** Primary outcome power analysis

P_1_(X = 0)	P_2_(X = 1)	P_2_-P_1_	β^*^	OR (x = 0 vs x = 1)	N (power = 80%)	Power (*N* = 600)
0.091	0.20	0.109	0.92	2.50	595	80.4%

Power is > 0.80 for detection of clinically meaningful effects of ≈ 10% to 11% group differences in treatment engagement.

#### Secondary outcomes: Analyses and power calculations for longitudinal analyses (over three waves) of depression and quality of life outcomes, treated as continuous, adjusting for the design effects of cluster randomization of churches

The basic regression model is: *y*_*ik*_ = *β*_0_ + *βxt*_*k*_ + *e*_*ik*_ (with *i* for subject, *k* for 3-time points, and *x* as a dummy variable for group). Change over the 3 waves of data (baseline, 3 months, and 6 months) will be modeled using a mixed multilevel model adjusting for clustering within churches. Power analyses were conducted assuming a cluster size of 20, and ICC of 0.03. Two scenarios regarding the average correlation between outcomes over time were examined (0.5 and 0.6). Assumptions related to the standard deviation were given above. Power calculations for sensitivity analyses relaxing the assumption of linear change were also performed, examining average change over time.

The power analysis equations for modeling rate of change as linear are as follows:

$n=\frac{2{\left({z}_{\alpha }+{z}_{\beta}\right)}^2{\sigma}^2\left(1-\rho \right){v}_{ifc}}{T_n{s}_x^2{d}^2{R}_{el}}$ and ${s}_x^2=\sum {\left({t}_j-\overline{t}\right)}^2/{T}_n=0.50/3$ [68]. The assumptions were: ρ = 0.5 (average correlation of outcomes over time), σ = (4.0,10), α = 0.05, R_el_ = 0.9 (reliability) and 3 time points (baseline, 3 months, 6 months) for most participants, with an n of 300 per group (20 per church) under ITT and 240 with attrition. Adjusting for clustering within church: V_ifc_ = 1.57, it will be possible to detect an effect size of δ = 1.2 (QIDS), δ = 3.00 (PROMIS depression/ poor QoL reduction (SF-12)) points per half year (Cohen’s d = 0.30), with power of 0.80. This translates to a group difference between usual care and intervention in QIDS of 1.20 points, or PROMIS Depression and SF-12 scores of 3.00 points at study end (after 6 months of follow-up). The following table (Table 4) is for QIDS, PROMIS depression or SF-12, and shows the net reduction (Cohen’s d) in QIDS, PROMIS depression, or poor QoL between usual care and intervention groups*.* As shown, relatively small effect sizes are detectable, within or below the range of clinically meaningful differences, even with missing data.

**Table 4:** Secondary Outcomes Effect Sizes: Modeling Rate of Change as Linear

*ρ* (average correlation)	QIDS (Cohen’s *d*) *n* = 300; *n* = 240 per group	PROMIS depression/ poor QoL reduction (Cohen’s *d*) *n* = 300; *n* = 240 per group
*ρ* = 0.5	1.20 (0.30) 1.30 (0.325)	3.00 (0.30) 3.25 (0.325)
*ρ* = 0.6	1.08 (0.27) 1.16 (0.290)	2.70 (0.27) 2.90 (0.290)

The power analysis equations for modeling rate of change as nonlinear are as follows:

$m=\frac{2{\left({z}_{\alpha }+{z}_{\beta}\right)}^2{\sigma}^2\left\{1+\left({T}_n-1\right)\rho \right\}{V}_{ifc}}{T_n{d}^2{R}_{el}}$ [2]. Assuming: *ρ* = 0.5 (average correlation of outcomes over time), *σ* = (4.0,10.00), *α* = 0.05, *R*_el_ = 0.9 (reliability), and 2 time points (3 months, 6 months, assuming no baseline group differences) for most participants, with an *n* of 300 per group (20 per church), and adjusting for clustering within church (*V*_if_ = 1.57), it will be possible to detect an effect size of *δ* = 1.048 (QIDS), *δ* = 2.62 (depression/ poor QoL reduction (SF-12)) points per half year (Cohen’s *d* = 0.262), with power of 0.80. This translates to an average difference (over two follow-up waves) in QIDS of 1.048 points, PROMIS depression, and SF-12 scores of 2.62 points between usual care and intervention groups. The following table (Table 5) shows QIDS, PROMIS depression, and poor QoL reduction (Cohen’s *d*) differences between usual care and intervention groups under the assumptions given above. For example, if due to imbalance or non-random patterns of missing data, the *n* is reduced by as much as 20% to 240, the average difference (over two follow-up waves) in PROMIS depression scores between usual care and intervention groups would be 2.81 points per half year (Cohen’s *d* = 0.281), a relatively small effect size. Other values are given in the table.

**Table 5:** Secondary outcome effect sizes: modeling rate of change as nonlinear

*ρ* (average correlation)	QIDS Depression (Cohen’s *d*) *n* = 300; *n = 240 per group*	PROMIS Depression/poor QoL reduction (Cohen’s *d*) *n* = 300; *n = 240 per group*
*ρ* = 0.5	1.048 (0.262) 1.12 (0.281)	2.62 (0.262) 2.81 (0.281)
*ρ* = 0.6	1.08 (0.270) 1.16 (0.290)	2.70 (0.270) 2.90 (0.290)

### Interim analyses {21b}

There are no pre-planned interim analyses or predetermined stopping guidelines. Thus, there are no stopping rules and no pre-planned interim analyses.

### Methods for additional analyses (e.g., subgroup analyses) {20b}

#### Power for PHQ-9 screening measure

Assuming that the observed s.d. = 4.7 for the PHQ-9, and that *ρ* = 0.5 (average correlation of outcomes over time), *α* = 0.05, *R*_el_ = 0.9 (reliability) and 3 time points (baseline, 3 months, 6 months) for most participants, with an *n* of 300 per group and adjusting for clustering within church (*V*_if_ = 1.57), it will be possible to detect an effect size of *δ* = 1.41 points per half year (Cohen’s *d* = 0.30) using the linear method, and an effect size of *δ* = 1.23 points per half year (Cohen’s *d* = 0.262) using the nonlinear method, with power of 0.80. The following table (Table 6) is for PHQ-9 reduction (Cohen’s *d*) between usual care and intervention groups, assuming *α* = 0.05, *R*_el_ = 0.9 (reliability), *σ* = 10, power = 0.80, with *ρ* = 0.5 or *ρ* = 0.6, *n* = 300 (20 per church), *V*_if_ = 1.57. Even with attrition, detectable effect sizes are relatively small with either a linear or nonlinear model (Cohen’s *d* from 0.27 to 0.33).

**Table 6:** PHQ-9 screening measure effect sizes

*ρ* (average correlation)	Linear method *n* = 300; *n* = 240 per group	Nonlinear method *n* = 300; *n* = 240 per group
*ρ* = 0.5	1.41 (0.30) 1.53 (0.325)	1.23 (0.262) 1.33 (0.281)
*ρ* = 0.6	1.27 (0.27) 1.36 (0.290)	1.27 (0.270) 1.37 (0.290)

In summary, given the above assumptions, a sample size of 300 per group is adequate for detection of relatively small intervention effects in secondary outcomes.

### Methods in analysis to handle protocol non-adherence and any statistical methods to handle missing data {20c}

Data will be collected at baseline, 3 months, and 6 months. Based on our previous studies in churches, we observed a 3-month attrition rate of 8%, and an overall cumulative 6-month attrition rate of 17 to 18%. The primary analyses will be conducted using an *intent-to-treat* approach. However, it is possible that analyses may include covariates if imbalance is observed. In the case of larger amounts of missing data than anticipated, using the maximum-likelihood multilevel modeling approach described below to estimate treatment effects, we will include the baseline data for these subjects in the analysis. Under the assumption that the missing data are either Missing Completely at Random or Missing at Random, this method, in conjunction with the covariate to adjust for attrition bias (if necessary), yields intent-to-treat parameter estimates that are consistent with what would be expected if there were no missing data. Although missing data can be treated within the mixed model if missing is at random, depending on the extent and nature of missing data, SAS Proc MI and MIANALYZE may be used. Space does not permit a more detailed description of the process.

### Plans to give access to the full protocol, participant level-data, and statistical code {31c}

The full protocol is available on clinicaltrials.gov—NCT04524767. The datasets analyzed during the current study will be available from the corresponding author on reasonable request.

## Oversight and monitoring

### Composition of the coordinating center and trial steering committee {5d}

The coordinating center and trial steering committee are composed of the Contact-Principal Investigator (Columbia University, Dept. of Psychiatry) and the Co-Principal Investigator (Co-PI) (Columbia University, Dept. of Neurology). The Contact-Principal Investigator (Columbia University, Dept. of Psychiatry) will be responsible for corresponding with NIMH. In addition to providing timely reports to NIMH, regarding (i) unanticipated problems or unexpected serious adverse events that may be related to the study protocol, (ii) IRB-approved revisions to the study protocol that indicate a change in risk for participants, and (iii) notice of any actions taken by the IRB or regulatory bodies regarding the research and any responses to those actions, the Contact-PI will be responsible for the following: reviewing all PHQ-9 questionnaires and assessments by participants students at baseline, 3 months, and 6 months; reviewing safety plans documented by CHWs; and reviewing rates of participant referral to treatment.

The Co-Principal Investigator (Co-PI) (Columbia University, Dept. of Neurology) will provide site oversight by auditing CHW protocols and church sites where the intervention and control programs will take place. In addition to intervention fidelity monitoring, the Co-PI will perform random interviews with CHWs (at least once at each church site) to evaluate the presence of any adverse reactions to the curriculum that may not be captured by questionnaire data.

The Project Director (PD) will be present on site at every intervention and control program. The PD will be responsible for identifying and reporting any adverse encounters—related or unrelated to the intervention—to the Co-PIs. These include adverse emotional responses, interpersonal conflicts, physical accidents, or any other participant safety concerns that may occur during the intervention.

This study has partnered with data coordinating center (DCC), an organization that is experienced in coordinating clinical trials and will be responsible for the following activities: (1) development of the computer-assisted data collection system, (2) staff training and certification in data collection, (3) randomization procedures, (4) data monitoring and quality control, (5) data processing, and (6) data analysis. The DCC will prepare regular reports for internal and external monitoring of progress toward study milestones and provide blinded and unblinded data requests.

### Composition of the data monitoring committee, its role and reporting structure {21a}

#### Data safety monitoring plan

In compliance with NIH requirements, we will establish a data and safety monitoring plan (DSMP). The purpose of these plans is to ensure the safety of participants and the validity and integrity of the data. Considering the study rationale, population, procedures, and the risk: benefit profile as outlined; the overall risk level for participation in this screening intervention is classified as minimal. Due to the classification of this study as minimal risk, the following members of the investigative team will serve as the Data Safety Monitoring Committee and will perform the monitoring: The Contact-Principal Investigator (Columbia University, Dept. of Psychiatry) will be responsible for corresponding with NIMH. In addition to providing timely reports to NIMH, regarding (i) unanticipated problems or unexpected serious adverse events that may be related to the study protocol, (ii) IRB-approved revisions to the study protocol that indicate a change in risk for participants, and (iii) notice of any actions taken by the IRB or regulatory bodies regarding the research and any responses to those actions, the Contact-PI will be responsible for the following: reviewing all PHQ-9 questionnaires and assessments by participants at baseline, 3 months, and 6 months; reviewing safety plans documented by CHWs; and reviewing rates of participant referral to treatment.

The Co-Principal Investigator (Co-PI) (Columbia University, Dept. of Neurology) will provide site oversight by auditing CHW protocols and church sites where the intervention and control programs will take place. In addition to intervention fidelity monitoring, the Co-PI will perform random interviews with CHWs (at least once at each church site) to evaluate the presence of any adverse reactions to the intervention that may not be captured by questionnaire data.

The Data Coordinating Center (DCC) will regularly review program data and discuss with the MPIs monthly. These data will include questionnaire items designed to capture adverse participant emotional responses to the intervention.

The Project Director (PD) will be present on site at every intervention and control program. The PD will be responsible for identifying and reporting any adverse encounters—related or unrelated to the intervention—to the Co-PIs. These include adverse emotional responses, interpersonal conflicts, physical accidents, or any other participant safety concerns that may occur during the intervention.

### Adverse event reporting and harms {22}

**Fig. 2:**
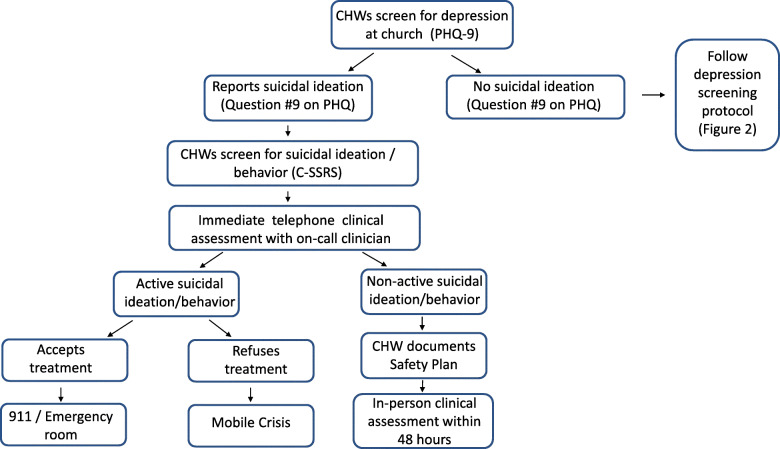
Safety assessment protocol

If a subject responds positively to Question #9 of the PHQ-9 or verbally endorses suicidality, the CHW will immediately initiate a safety assessment protocol (Fig. 2). The CHW will assess risk with the Columbia-Suicide Severity Rating Scale (C-SSRS). After administering the C-SSRS, the CHW will immediately call the on-call study clinician (Co-PIs or another licensed clinician). The on-call clinician will then conduct a brief, standardized clinical interview that reviews the score on the C-SSRS and quantifies the specific suicidal plans and access to lethal means (e.g., firearms). For participants with *active* suicidal ideation or behavior with imminent risk of harm to self or others, severe disability, or psychosis, the CHW will immediately collaborate with church staff and the participant’s family for transfer to a local Emergency Room or Mobile Crisis. For participants with *non-active* suicidal ideation/behavior, the CHW will inform the study PIs within 24 h and complete the Stanley-Brown Safety Plan [69, 70]. The CHW will inquire to see if the subject is currently in treatment, and if so, he/she will attempt to contact the participant’s mental health provider. The subject will be referred for an in-person clinical interview with the on-call study clinician within 48 h. If the subject does not attend or refuses the in-person assessment, Mobile Crisis will be dispatched to the residence.

### Frequency and plans for auditing trial conduct {23}

#### Monitoring study safety

The data and safety monitoring plan will consist of reporting of adverse events to the IRB and to NIH trials. Adverse events will be reported to the Columbia IRB, which has the authority to halt the trial if it perceives that harm is occurring due to the intervention. Summaries of adverse events reports will be made to NIH in the yearly progress report and at the end of year 5, in the final report, unless the nature of a particular event is such that it bears reporting to NIH immediately. The progress of the trial will also be evaluated from the initial screening of participants by inclusion and exclusion criteria to the informed consent process to the provision of participant study instruction to staff training in Good Clinical Practices (GCP) and regulations pertaining to the Conduct of Human Participant Research. This will also include internal monthly quality control audits, periodic assessments of data quality and timeliness, participant recruitment, accrual and retention, and protocol fidelity monitoring. One or more “Early Safety/Trial Integrity Reviews” will be held during the early stage of protocol enrollment, to review early safety information, to review factors relating to quality of trial conduct, and to ensure proper implementation of procedures to reassess the sample size.

### Plans for communicating important protocol amendments to relevant parties (e.g., trial participants, ethical committees) {25}

Protocol amendments will be submitted to Columbia University’s IRB, and participants will be informed, if necessary. Annual progress reports will be submitted to the funder (NIMH). Any deviations from the protocol will be fully documented using a breach report form. The protocol will be updated in the clinical trial registry NCT04524767; clinicaltrials.gov.

### Dissemination plans {31a}

#### Community dissemination

The Community Coalition will organize a dissemination symposium at a community-based venue in Harlem. The symposium will be *co-presented* by the investigators and selected members of the Coalition and will be specifically organized to garner community feedback. The content of the symposium will be distributed to the participating churches and stakeholder organizations [71].

#### Study dissemination and implementation

If our SBIRT intervention is shown to be effective in this RCT, our next step will be to design a Hybrid Type 3 Implementation-Effectiveness Study to disseminate and implement the intervention across the vast population of Black Churches in New York State.

## Discussion

Depression is a leading cause of disability costing U.S. taxpayers $210 billion annually [72]. Significant racial disparities in depression exist with African American (AA) adults more disabled from the disease, and less likely to receive treatment compared to Whites [1, 2]. These disparities have been linked to socio-economic, cultural, and contextual factors governing under-detection and under-treatment of depression [3]. For example, although most depression screening and treatment occurs in primary care settings, AAs are half as likely to be screened for depression in these settings compared to white adults [13, 73]. The current proposal seeks to test a novel “meet people where they are” engagement model for depression screening and treatment referral among AAs, and evaluate its effect on detection of depression and receipt of treatment.

Churches are among the most trusted and influential institutions within AA communities [74]. AAs have the highest rates of church attendance among all racial/ethnic groups in the USA, with over 60% attending church several times per month [75]. Approximately 72% of AAs with a serious personal problem, including depression, seek help in Black churches [35]. Indeed, in our prior work, we found that 20% of adults in Black churches screened positive for depression using the Patient Health Questionnaire-9 (PHQ-9) [76]. However, subjects with a positive depression screen (PHQ-9 ≥ 10) universally declined treatment referral when offered by research coordinators [77]. Importantly, and relevant to the current application, a significant knowledge gap exists regarding effective strategies for linking church-based depression screening to engagement with clinical providers among AAs [78].

Community Health Workers (CHWs) are trusted, culturally concordant lay health personnel from the local community with significant social capital [79]. CHWs have proven effective at providing evidence-based screening and linkages to medical care for several chronic illnesses such as cancer and cardiovascular disease [80]. Although these conditions do not carry the same level of stigma as depression among AAs, we hypothesize that CHWs deployed for church-based depression screening can help overcome cognitive barriers and increase treatment engagement—defined as attending a depression-related clinical visit for which the subject reported receiving information, referral, counseling, or medication for depression [54].

This proposal builds on extensive experience with depression and church-based research by the study team at Columbia University. The Co-PI completed a NIH-funded Randomized Controlled Trial targeting care-seeking behaviors for acute stroke, which involved 312 Black and Hispanic adults from 13 churches (Williams et al., 2019, JAMA Neurology) [81]. In a separate NIMH-funded study (K23-MH102540), The Contact-PI trained 263 AA church members in an evidence-based mental health literacy intervention [82]. Additionally, through intramural funding, the Co-PIs created an 8-week CHW training program for Black churches in Harlem, NY, which includes the evidence-based, SBIRT approach [83].

## Trial status

Protocol version IRB-AAAT1474; July 27, 2020. Recruitment for this study will begin in May 2021 and the approximate recruitment completion date will be in 2025.

## Data Availability

Analyses will be conducted by the Data Coordinating Center. Plans for data archiving and sharing will be made within 2 years of study completion. Guidelines for data sharing will be followed.

## Abbreviations

AA: African American
CHW: Community health worker
CFIR: Consolidated Framework for Implementation Research
C-SSRC: Columbia-Suicide Severity Rating Scale
DCC: Data Coordinating Center
MI: Motivational interviewing
PC: Project Coordinator
PD: Project Director
PHQ-9: Patient Health Questionnaire-9
QIDS-SR: Quick Inventory of Depressive Symptomatology
QoL: Mental Health-Related Quality of Life
RA: Research assistant
RAU: Referral as usual
RCT: Randomized controlled trial
SBIRT: Screening Brief Intervention and Referral to Treatment
